# Synthesis and structure determination of racemic (Δ/Λ)-tris­(ethyl­enedi­amine)­cobalt(III) trichloride hemi(hexa­aqua­sodium chloride)

**DOI:** 10.1107/S2056989021009336

**Published:** 2021-09-14

**Authors:** Linden H. Conrad-Marut, Eric W. Reinheimer, Adam R. Johnson

**Affiliations:** a Department of Chemistry, Harvey Mudd College, 301 Platt Avenue, Claremont, CA 91711-5990, USA; b Rigaku Americas Corporation, 9009 New Trails Dr., The Woodlands, TX 77381, USA

**Keywords:** crystal structure, racemate, enanti­omer, ethyl­ene di­amine

## Abstract

The synthesis and crystal structure of the title racemic compound, [Co(C_2_H_8_N_2_)_3_]Cl_3_.{[Na(H_2_O)_6_]Cl}_0.5_, are reported. The trivalent cobalt atom, which resides on a crystallographic threefold axis, is chelated by a single ethyl­ene di­amine (en) ligand and yields the tris-chelate [Co(en)_3_]^3+^ cation with distorted octa­hedral geometry after the application of crystal symmetry. The sodium cation (site symmetry ), has a single water mol­ecule bound to it in the asymmetric unit and yields a distorted, octa­hedrally coordinated hydrated [Na(H_2_O)_6_]^+^ cation after the application of symmetry. One of the chloride ions lies on a general position and the other has  site symmetry. An extensive array of C—H**⋯**O, N—H**⋯**Cl and O—H**⋯**Cl hydrogen bonds exists between the ethyl­ene di­amine ligands, the water mol­ecules of hydration, and the anions present, thereby furnishing solid-state stability.

## Abstract 2

In the racemic title compound, the [Co(en)_3_]^3+^ and [Na(H_2_O)_6_]^+^cations exist in distorted octa­hedral coordination environments and charge neutrality in the salt is furnished by Cl^−^ anions. Structural cohesion is maintained by an array of C—H**⋯**O, N—H**⋯**Cl and O—H**⋯**Cl hydrogen bonds.

## Chemical context   

The coordination complex-cation tris-ethyl­enedi­amine cobalt(III), [Co(en)_3_]^3+^, was influential in Werner’s development of the structure of transition-metal complexes as it could be resolved into its two enanti­omers by selective crystallization using tartrate anions, thus helping to demonstrate the octa­hedral geometry of the metal ion (Werner, 1912 ▸). As such, the synthesis of members of this family of complexes is a common undergraduate laboratory experiment (Work & McReynolds, 1946 ▸; Broomhead *et al.*, 1960 ▸; Girolami *et al.*, 1999 ▸; McClellan & Cass, 2015 ▸).

The synthesis and structural characterization of many members of this family of complexes, both racemic and resolved, have been undertaken over the years. In all cases, the [Co(en)_3_]^3+^ complex cation was found to have trigonally distorted octa­hedral symmetry, as expected, and the structures usually have significant hydrogen-bonding inter­actions involving the ethyl­ene di­amine ligands, the water mol­ecules of hydration, and the anions present.
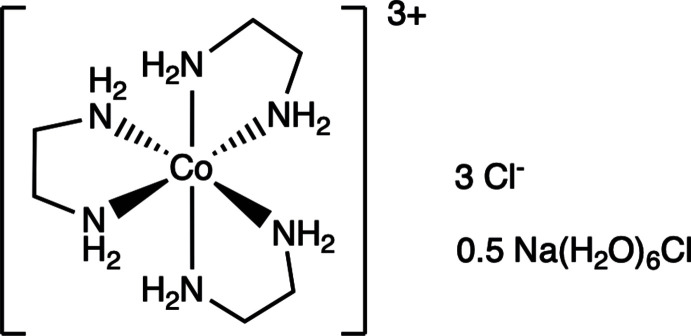


## Structural commentary   

**Figure 1:**
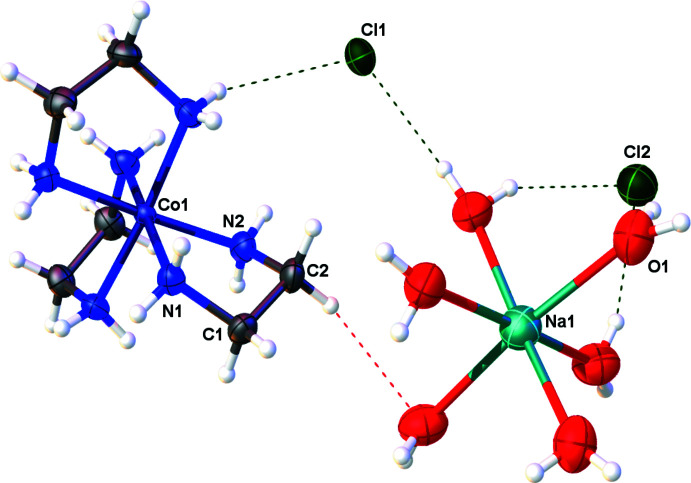
Anisotropic displacement ellipsoid plot of **1** with ellipsoids set to the 50% probability level. Atoms in the asymmetric unit are labeled. Dashed lines represent hydrogen bonds.

The title compound crystallizes in the centrosymmetric trigonal space group *P* 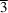 *c1* (Fig. 1 ▸). The asymmetric unit consists of a trivalent cobalt atom residing on a threefold axis chelated by an ethyl­ene di­amine (en; C_2_H_8_N_2_) ligand. Two Cl anions, one occupying a general position and the other lying on a 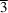 axis are also present. One Na cation, also positioned on a 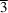 axis, with a water mol­ecule (general position) bound to it are also observed. After application of crystal symmetry, the [Co(en)_3_]^3+^ and [Na(H_2_O)_6_]^+^ cationic complexes that result each adopt distorted octa­hedral geometries.

Within the chelating en ligand, given the *sp^3^*-hybridization of the C atoms and an expected tetra­hedral coordination environment around those C atoms, bond angles around each should be near the expected 109.5°. The values obtained from the crystal structure [minimum = 106.33 (15)°; maximum = 111.2 (12)°] indicate a degree of distortion.

Solid-state integrity is maintained by an array of C—H**⋯**O, N—H**⋯**Cl and O—H**⋯**Cl hydrogen bonds between the [Co(en)_3_]^3+^ and [Na(H_2_O)_6_]^+^cations and the chloride anions. Unlike the structure of enanti­opure Λ-[tris­(ethyl­enedi­amine)­cobalt(III) trichloride]·0.5NaCl·3H_2_O (Nakatsu *et al.*, 1957 ▸; Farrugia *et al.*, 2000 ▸), where the sodium cations and chloride anions showed signs of disorder, no features suggestive of disorder are observed in the structure of the racemate.

## Supra­molecular features   

The en-chelated, trivalent cobalt atom in the title compound lies on a threefold axis housed within the (021) plane. As a result of crystal symmetry, the full [Co(en)_3_]^3+^ cation is generated and shows both the Λδδδ and the Δλλλ configurations with distorted octa­hedral geometry (Jensen, 1970 ▸). By virtue of its residing on a threefold axis, the net +1 charge that results from the Co atom is balanced by a fully occupied Cl anion occupying a general position. Typically, changes in conformation of the en ligand can be attributed to hydrogen bonding; however, all en conformations in both Co(en)_3_ and Cr(en)_3_ cations demonstrate similar energies (Veal & Hodgson, 1972 ▸; Enemark *et al.*, 1970 ▸; Raymond *et al.*, 1968*a* ▸,*b* ▸; Raymond & Ibers, 1968 ▸). Analogous to the many structures encompassing the [Co(en)_3_]^3+^ cation, hydrogen-bonded arrays are prevalent in the solid-state structure between the en ligands and both water mol­ecules and chloride anions (Table 1 ▸).

**Figure 2:**
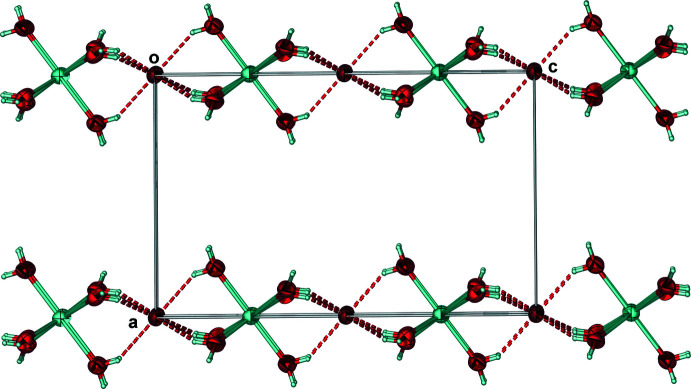
Face-sharing octa­hedra looking into the *ac* plane encompassing the Na(H_2_O)_6_^+^ cations and six water mol­ecules hydrogen bonded to Cl2 that form a herringbone pattern with respect to one another. Anisotropic displacement ellipsoids have been set to the 50% probability level. Dashed lines represent hydrogen bonds.

**Figure 3:**
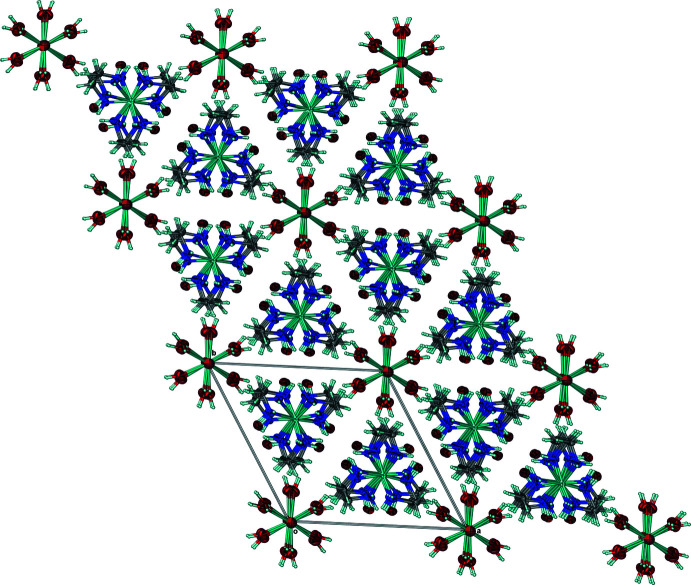
Packing of **1** relative to the *ab* plane. Collectively, the symmetry elements make the structure of **1** an excellent example of the *p6mm* two-dimensional space group. Anisotropic displacement ellipsoids have been set to the 50% probability level.

It is notable that Cl2 accepts six, symmetry-equivalent O1—H1*E*⋯Cl2 hydrogen bonds (Table 1 ▸) and forms a distorted Cl(H_2_O)_6_ octa­hedron. Along the *c*-axis, the orientation of the sodium and Cl2 octa­hedra with respect to one another forms a herringbone-type pattern when looking into the *ac* plane (Fig. 2 ▸). Collectively, the symmetry elements within the solid-state structure of the racemate make it an excellent illustration of the *p6mm* two-dimensional space group when looking towards the *ab* plane (Fig. 3 ▸).

## Database survey   

The structure of racemic [Co(en)_3_]Cl_3_ was reported to have the trigonal space group *P* 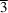 *c1*; however, no additional structural details were reported (Dingle & Ballhausen, 1967 ▸). This salt was later crystallized as the non-stoichiometric hydrate (2.8 water mol­ecules per cobalt center) with long chains of hydrogen-bonded water mol­ecules that precluded inter­actions between the incorporated water mol­ecules and the ethyl­ene di­amine ligands (Whuler *et al.*, 1975 ▸). This same salt was later crystallized as the tetra­hydrate and included a one-dimensional water chain perpendicular to the [001] direction. The solid was vacuum dried to form void channels that could incorporate guest mol­ecules (Takamizawa *et al.*, 2008 ▸).

Like racemic [Co(en)_3_]Cl_3_, other chemically-similar salts have been crystallized that demonstrate hydrogen-bonding arrays involving the ethyl­enedi­amine ligands, inter­stitial water mol­ecules, and the counter-ions. These included the Λ-enanti­omer of the monohydrate Cl and I salts, which crystallize in the tetra­gonal space group *P4_3_2_1_2*, in 1969 and 2001, respectively (Iwata *et al.*, 1969 ▸; Matsuki *et al.*, 2001 ▸). The structure of the bromide salt of the Δ-enanti­omer, Δ-[Co(en)_3_]Br_3_·H_2_O, was carried out in 1962, though the absolute structure could not be determined by anomalous dispersion at that time (Nakatsu, 1962 ▸). The structure of the right-handed helical enanti­omer, Δ-[Co(en)_3_]I_3_·H_2_O was finally reported in 2019 and crystallizes in the ortho­rhom­bic space group *P2_1_2_1_2_1_* (Grant *et al.*, 2019 ▸).

More complex counter-ions have also been utilized in racemic and purely enanti­omeric salts with [Co(en)_3_]^3+^. The structures of the nitrate salts, obtained both as a racemic crystal in the *Pca2_1_* space group (Oldenbourg, 1998 ▸) and as the Λ-enanti­omer in the *P4_1_2_1_2* space group (Witiak *et al.*, 1972 ▸) were reported. Racemic crystals [Co(en)_3_][Cr(CN)_5_(NO)]·2H_2_O (Enemark *et al.*, 1970 ▸) and [Co(en)_3_]_2_[CdCl_6_]Cl_2_·2H_2_O (Veal & Hodgson, 1972 ▸) were also reported, which crystallize in the monoclinic space group *P2_1_/c*. The complex racemic hydrogen phosphate salt [Co(en)_3_]_2_[HPO_4_]_3_·9H_2_O was determined to exist in the ortho­rhom­bic space group *Pnma* (Raymond & Duesler, 1971 ▸). In this latter-most structure, it has been proposed that the significant hydrogen bonding involving the en ligands, the counter-ion and the water mol­ecules of hydration is directly responsible for this material’s circular dichroism spectrum (Raymond & Duesler, 1971 ▸).

As the student laboratory preparation usually involves the synthesis of the racemic double salt [Co(en)_3_]·Cl_3_·0.5NaCl·3H_2_O (McClellan & Cass, 2015 ▸; Girolami *et al.*, 1999 ▸), we were surprised to not find its structure reported in the Cambridge Structural Database. As mentioned previously, the structure of the Λ-enanti­omer of the complex was first reported in 1957 (Nakatsu *et al.*, 1957 ▸) and later redetermined in 2000 (Farrugia *et al.*, 2000 ▸).

## Synthesis and crystallization   

The title complex **1** was prepared following the method of Girolami (Girolami *et al.*, 1999 ▸) and later modified by Cass (McClellan & Cass, 2015 ▸). Into a 100 ml beaker, CoCl_2_·6H_2_O (6.0 g, 25 mmol, finely ground using a mortar and pestle) was dissolved in water (20 mL) with stirring. Upon addition of ethyl­enedi­amine di­hydro­chloride (13.3 g, 100 mmol), the solution became pink and cloudy. Sodium hydroxide pellets (6.75 g, 170 mmol) were next added slowly while the solution stirred. Each pellet initially turned blue and then completely dissolved within a few minutes. The pH was then tested using litmus paper and determined to be 8. Hydro­chloric acid (6 *M*) was added dropwise until the pH was approximately 7–7.5, which changed the color of the solution to rusty orange. A hydrogen peroxide solution (20 ml of 3% solution) was added dropwise over a couple of minutes and the solution became dark orange. The solution was slowly brought to a boil while stirring. The stir bar was removed and the beaker placed into an ice bath for 30 minutes to cool. The crystals were collected through filtration and washed with 95% ethanol (50 ml) and subsequently diethyl ether (20 ml) to yield a bright-orange powder (6.754 g, 18.6 mmol, 74.5%). Large single crystals (*ca* 3 × 3 ×3 mm) of **1** were grown by slow evaporation from water and cut to size using a razor blade.

## Refinement   

Crystal data, data collection and structure refinement details for **1** are summarized in Table 2 ▸. With the exception of atom H1*E*, which was constrained to ride on the water O atom (O1), all other H atoms were located in the difference-Fourier map and freely refined with 0.91 < C—H < 0.99 Å, 0.81 < N—H < 0.84 Å, and O—H = 0.89 Å.

## Supplementary Material

Crystal structure: contains datablock(s) I. DOI: 10.1107/S2056989021009336/hb7980sup1.cif

Structure factors: contains datablock(s) I. DOI: 10.1107/S2056989021009336/hb7980Isup2.hkl

Supporting information file. DOI: 10.1107/S2056989021009336/hb7980Isup3.cml

CCDC reference: 2042981

Additional supporting information:  crystallographic information; 3D view; checkCIF report

## Figures and Tables

**Table 1:** Hydrogen-bond geometry (Å, °)

*D*—H⋯*A*	*D*—H	H⋯*A*	*D*⋯*A*	*D*—H⋯*A*
N1—H1*A*⋯Cl1^i^	0.84 (2)	2.57 (2)	3.3586 (13)	156.7 (18)
N1—H1*B*⋯Cl1^ii^	0.81 (2)	2.75 (2)	3.4317 (13)	142.0 (19)
N2—H2*A*⋯Cl1^iii^	0.84 (2)	2.50 (2)	3.3286 (14)	172 (2)
N2—H2*B*⋯Cl1	0.82 (2)	2.62 (2)	3.2925 (13)	140.6 (19)
O1—H1*E*⋯Cl2	0.88	2.35	3.1354 (18)	147
O1—H1*F*⋯Cl1	0.89 (4)	2.41 (4)	3.2787 (18)	164 (3)
C2—H2*D*⋯O1^iv^	0.92 (2)	2.59 (2)	3.361 (2)	142.1 (17)

Symmetry codes: (i) x-y+1, -y+1, -z+{\script{3\over 2}}; (ii) x, x-y+1, z+{\script{1\over 2}}; (iii) -x, -x+y, -z+{\script{3\over 2}}; (iv) y-1, x, -z+{\script{3\over 2}}.

**Table 2:** Experimental details

Crystal data	
Chemical formula	[Co(C_2_H_8_N_2_)_3_]Cl_3_·{[Na(H_2_O)_6_]Cl}_0.5_
*M*_r_	428.86
Crystal system, space group	Trigonal, *P*\overline{3}*c*1
Temperature (K)	293
*a*, *c* (Å)	11.4290 (2), 15.5815 (2)
*V* (Å^3^)	1762.61 (7)
*Z*	4
Radiation type	Mo *K*α
μ (mm^−1^)	1.53
Crystal size (mm)	0.39 × 0.29 × 0.22

Data collection	
Diffractometer	XtaLAB Mini II
Absorption correction	Analytical (*CrysAlis PRO*; Rigaku OD, 2020 ▸)
*T*_min_, *T*_max_	0.664, 0.794
No. of measured, independent and observed [*I* > 2σ(*I*)] reflections	65362, 1860, 1643
*R*_int_	0.027
(sin θ/λ)_max_ (Å^−1^)	0.722

Refinement	
*R*[*F*^2^ > 2σ(*F* ^2^)], *wR*(*F* ^2^), *S*	0.027, 0.072, 1.06
No. of reflections	1860
No. of parameters	99
H-atom treatment	H atoms treated by a mixture of independent and constrained refinement
Δρ_max_, Δρ_min_ (e Å^−3^)	0.47, −0.76

Computer programs: *CrysAlis PRO* (Rigaku OD, 2020 ▸), *SHELXT* (Sheldrick, 2015*a* ▸), *SHELXL* (Sheldrick, 2015*b* ▸), and *OLEX2* (Dolomanov *et al.*, 2009 ▸).

## supplementary crystallographic information

### Crystal data

**Table:**

[Co(C_2_H_8_N_2_)_3_]Cl_3_·{[Na(H_2_O)_6_]Cl}_0.5_	*D*_x_ = 1.616 Mg m^−^^3^
*M**_r_* = 428.86	Mo *K*α radiation, λ = 0.71073 Å
Trigonal, *P*3*c*1	Cell parameters from 30262 reflections
*a* = 11.4290 (2) Å	θ = 2.1–30.7°
*c* = 15.5815 (2) Å	µ = 1.53 mm^−^^1^
*V* = 1762.61 (7) Å^3^	*T* = 293 K
*Z* = 4	Block, clear light orange
*F*(000) = 896	0.39 × 0.29 × 0.22 mm

### Data collection

**Table:**

XtaLAB Mini II diffractometer	1860 independent reflections
Radiation source: fine-focus sealed X-ray tube, Rigaku (Mo) X-ray Source	1643 reflections with *I* > 2σ(*I*)
Graphite monochromator	*R*_int_ = 0.027
Detector resolution: 10.0000 pixels mm^-1^	θ_max_ = 30.9°, θ_min_ = 2.6°
ω scans	*h* = −16→16
Absorption correction: analytical (CrysalisPro; Rigaku OD, 2020)	*k* = −16→16
*T*_min_ = 0.664, *T*_max_ = 0.794	*l* = −22→22
65362 measured reflections	

### Refinement

**Table:**

Refinement on *F*^2^	Primary atom site location: dual
Least-squares matrix: full	Hydrogen site location: difference Fourier map
*R*[*F*^2^ > 2σ(*F*^2^)] = 0.027	H atoms treated by a mixture of independent and constrained refinement
*wR*(*F*^2^) = 0.072	*w* = 1/[σ^2^(*F*_o_^2^) + (0.0373*P*)^2^ + 0.9272*P*] where *P* = (*F*_o_^2^ + 2*F*_c_^2^)/3
*S* = 1.06	(Δ/σ)_max_ = 0.001
1860 reflections	Δρ_max_ = 0.47 e Å^−^^3^
99 parameters	Δρ_min_ = −0.76 e Å^−^^3^
0 restraints	

### Special details

**Table:**

Geometry. All esds (except the esd in the dihedral angle between two l.s. planes) are estimated using the full covariance matrix. The cell esds are taken into account individually in the estimation of esds in distances, angles and torsion angles; correlations between esds in cell parameters are only used when they are defined by crystal symmetry. An approximate (isotropic) treatment of cell esds is used for estimating esds involving l.s. planes.

### Fractional atomic coordinates and isotropic or equivalent isotropic displacement parameters (Å^2^)

**Table:**

	*x*	*y*	*z*	*U*_iso_*/*U*_eq_	
Co1	0.3333	0.6667	0.88151 (2)	0.01756 (9)	
Cl1	0.10242 (4)	0.61265 (4)	0.62517 (2)	0.03423 (11)	
Cl2	0.0000	1.0000	0.5000	0.0546 (3)	
Na1	0.0000	1.0000	0.7500	0.0543 (5)	
N1	0.29983 (13)	0.51120 (12)	0.95321 (8)	0.0244 (2)	
N2	0.18593 (13)	0.53380 (13)	0.80958 (8)	0.0273 (2)	
O1	0.08849 (18)	0.88917 (17)	0.65619 (12)	0.0592 (4)	
H1E	0.0758	0.9001	0.6015	0.089*	
C1	0.17218 (16)	0.39077 (15)	0.92577 (10)	0.0302 (3)	
C2	0.16421 (17)	0.39746 (16)	0.82971 (10)	0.0316 (3)	
H2C	0.235 (2)	0.386 (2)	0.8014 (13)	0.036 (5)*	
H1A	0.363 (2)	0.494 (2)	0.9459 (13)	0.036 (5)*	
H1C	0.171 (2)	0.310 (2)	0.9475 (15)	0.042 (6)*	
H1D	0.103 (2)	0.396 (2)	0.9513 (13)	0.033 (5)*	
H2D	0.081 (2)	0.332 (2)	0.8111 (13)	0.040 (6)*	
H2A	0.114 (2)	0.533 (2)	0.8215 (14)	0.038 (5)*	
H1B	0.291 (2)	0.519 (2)	1.0044 (14)	0.038 (5)*	
H2B	0.198 (2)	0.546 (2)	0.7578 (14)	0.042 (6)*	
H1F	0.105 (4)	0.824 (4)	0.639 (2)	0.096 (11)*	

### Atomic displacement parameters (Å^2^)

**Table:**

	*U*^11^	*U*^22^	*U*^33^	*U*^12^	*U*^13^	*U*^23^
Co1	0.01928 (11)	0.01928 (11)	0.01412 (13)	0.00964 (5)	0.000	0.000
Cl1	0.03216 (19)	0.0423 (2)	0.02759 (18)	0.01808 (16)	0.00203 (13)	0.00448 (14)
Cl2	0.0568 (5)	0.0568 (5)	0.0501 (7)	0.0284 (2)	0.000	0.000
Na1	0.0515 (7)	0.0515 (7)	0.0598 (13)	0.0258 (4)	0.000	0.000
N1	0.0287 (6)	0.0247 (5)	0.0207 (5)	0.0141 (5)	−0.0015 (4)	0.0011 (4)
N2	0.0278 (6)	0.0296 (6)	0.0222 (5)	0.0126 (5)	−0.0053 (4)	−0.0026 (4)
O1	0.0636 (10)	0.0485 (9)	0.0674 (10)	0.0295 (8)	−0.0137 (8)	−0.0076 (8)
C1	0.0318 (7)	0.0225 (6)	0.0299 (7)	0.0088 (5)	0.0024 (5)	0.0017 (5)
C2	0.0340 (7)	0.0246 (6)	0.0289 (7)	0.0092 (6)	−0.0045 (6)	−0.0067 (5)

### Geometric parameters (Å, º)

**Table:**

Co1—N1^i^	1.9676 (12)	N1—H1A	0.84 (2)
Co1—N1^ii^	1.9676 (12)	N1—H1B	0.81 (2)
Co1—N1	1.9677 (12)	N2—C2	1.484 (2)
Co1—N2^i^	1.9601 (12)	N2—H2A	0.84 (2)
Co1—N2^ii^	1.9601 (12)	N2—H2B	0.82 (2)
Co1—N2	1.9600 (12)	O1—H1E	0.8837
Na1—O1^iii^	2.4586 (19)	O1—H1F	0.89 (4)
Na1—O1^iv^	2.4586 (19)	C1—C2	1.504 (2)
Na1—O1^v^	2.4587 (19)	C1—H1C	0.97 (2)
Na1—O1	2.4586 (19)	C1—H1D	0.91 (2)
Na1—O1^vi^	2.4586 (19)	C2—H2C	0.99 (2)
Na1—O1^vii^	2.4586 (19)	C2—H2D	0.92 (2)
N1—C1	1.4825 (19)		
			
N1^i^—Co1—N1^ii^	90.94 (5)	O1^iii^—Na1—O1^v^	96.03 (8)
N1^i^—Co1—N1	90.94 (5)	Co1—N1—H1A	109.1 (14)
N1^ii^—Co1—N1	90.94 (5)	Co1—N1—H1B	115.9 (15)
N2^i^—Co1—N1^i^	85.48 (5)	C1—N1—Co1	109.36 (9)
N2—Co1—N1^ii^	174.50 (5)	C1—N1—H1A	108.0 (14)
N2—Co1—N1	85.48 (5)	C1—N1—H1B	105.5 (16)
N2—Co1—N1^i^	93.29 (6)	H1A—N1—H1B	109 (2)
N2^i^—Co1—N1	174.50 (5)	Co1—N2—H2A	109.7 (15)
N2^ii^—Co1—N1^i^	174.50 (6)	Co1—N2—H2B	115.3 (15)
N2^ii^—Co1—N1	93.29 (6)	C2—N2—Co1	108.69 (9)
N2^i^—Co1—N1^ii^	93.29 (6)	C2—N2—H2A	107.2 (16)
N2^ii^—Co1—N1^ii^	85.48 (5)	C2—N2—H2B	108.5 (15)
N2^i^—Co1—N2^ii^	90.55 (6)	H2A—N2—H2B	107 (2)
N2^i^—Co1—N2	90.55 (6)	Na1—O1—H1E	111.2
N2—Co1—N2^ii^	90.55 (6)	Na1—O1—H1F	156 (2)
O1^iv^—Na1—O1^v^	88.27 (6)	H1E—O1—H1F	87.3
O1^iv^—Na1—O1	96.03 (8)	N1—C1—C2	107.24 (12)
O1—Na1—O1^v^	174.05 (8)	N1—C1—H1C	108.8 (14)
O1^vi^—Na1—O1^iv^	174.05 (8)	N1—C1—H1D	107.4 (13)
O1^vi^—Na1—O1^v^	87.75 (8)	C2—C1—H1C	115.0 (13)
O1^vii^—Na1—O1^iv^	88.27 (6)	C2—C1—H1D	110.8 (13)
O1^vii^—Na1—O1	87.75 (8)	H1C—C1—H1D	107.4 (18)
O1^vi^—Na1—O1^iii^	88.27 (6)	N2—C2—C1	106.33 (12)
O1^iii^—Na1—O1	88.27 (6)	N2—C2—H2C	109.2 (13)
O1^vii^—Na1—O1^iii^	174.05 (8)	N2—C2—H2D	110.3 (13)
O1^vii^—Na1—O1^v^	88.27 (6)	C1—C2—H2C	111.2 (12)
O1^iv^—Na1—O1^iii^	87.75 (9)	C1—C2—H2D	109.8 (13)
O1^vi^—Na1—O1	88.27 (6)	H2C—C2—H2D	110.0 (18)
O1^vi^—Na1—O1^vii^	96.03 (8)		

Symmetry codes: (i) −*x*+*y*, −*x*+1, *z*; (ii) −*y*+1, *x*−*y*+1, *z*; (iii) −*y*+1, *x*−*y*+2, *z*; (iv) *x*−*y*+1, −*y*+2, −*z*+3/2; (v) *y*−1, *x*+1, −*z*+3/2; (vi) −*x*+*y*−1, −*x*+1, *z*; (vii) −*x*, −*x*+*y*, −*z*+3/2.

### Hydrogen-bond geometry (Å, º)

**Table:**

*D*—H···*A*	*D*—H	H···*A*	*D*···*A*	*D*—H···*A*
N1—H1*A*···Cl1^viii^	0.84 (2)	2.57 (2)	3.3586 (13)	156.7 (18)
N1—H1*B*···Cl1^ix^	0.81 (2)	2.75 (2)	3.4317 (13)	142.0 (19)
N2—H2*A*···Cl1^vii^	0.84 (2)	2.50 (2)	3.3286 (14)	172 (2)
N2—H2*B*···Cl1	0.82 (2)	2.62 (2)	3.2925 (13)	140.6 (19)
O1—H1*E*···Cl2	0.88	2.35	3.1354 (18)	147
O1—H1*F*···Cl1	0.89 (4)	2.41 (4)	3.2787 (18)	164 (3)
C2—H2*D*···O1^x^	0.92 (2)	2.59 (2)	3.361 (2)	142.1 (17)

Symmetry codes: (vii) −*x*, −*x*+*y*, −*z*+3/2; (viii) *x*−*y*+1, −*y*+1, −*z*+3/2; (ix) *x*, *x*−*y*+1, *z*+1/2; (x) *y*−1, *x*, −*z*+3/2.
